# Extrinsic Calibration of Camera Networks Based on Pedestrians

**DOI:** 10.3390/s16050654

**Published:** 2016-05-09

**Authors:** Junzhi Guan, Francis Deboeverie, Maarten Slembrouck, Dirk Van Haerenborgh, Dimitri Van Cauwelaert, Peter Veelaert, Wilfried Philips

**Affiliations:** Image Processing and Interpretation, TELIN, Ghent University/iMinds, Sint Pietersnieuwstraat 41, 9000 Gent, Belgium; Francis.Deboeverie@telin.ugent.be (F.D.); Maarten.Slembrouck@telin.ugent.be (M.S.); dirk.vanhaerenborgh@telin.ugent.be (D.V.H.); Dimitri.VanCauwelaert@telin.ugent.be (D.V.C.); peter.veelaert@ugent.be (P.V.); philips@telin.UGent.be (W.P.)

**Keywords:** extrinsic calibration, camera network, pedestrians, orthogonal Procrustes

## Abstract

In this paper, we propose a novel extrinsic calibration method for camera networks by analyzing tracks of pedestrians. First of all, we extract the center lines of walking persons by detecting their heads and feet in the camera images. We propose an easy and accurate method to estimate the 3D positions of the head and feet w.r.t. a local camera coordinate system from these center lines. We also propose a RANSAC-based orthogonal Procrustes approach to compute relative extrinsic parameters connecting the coordinate systems of cameras in a pairwise fashion. Finally, we refine the extrinsic calibration matrices using a method that minimizes the reprojection error. While existing state-of-the-art calibration methods explore epipolar geometry and use image positions directly, the proposed method first computes 3D positions per camera and then fuses the data. This results in simpler computations and a more flexible and accurate calibration method. Another advantage of our method is that it can also handle the case of persons walking along straight lines, which cannot be handled by most of the existing state-of-the-art calibration methods since all head and feet positions are co-planar. This situation often happens in real life.

## 1. Introduction

Visual surveillance systems (VSS) have a wide variety of applications in numerous aspects of life. Camera calibration is an important topic in visual surveillance. It is the essential step for 3D position detection, tracking and further video content analysis tasks. The parameters of a camera to be calibrated are divided into two classes: Intrinsic and extrinsic. The intrinsic parameters define the imaging geometry and the optical characteristics of each camera individually. The extrinsic parameters denote the coordinate system transformations from 3D world coordinates to 3D camera coordinates. While intrinsic parameters usually need to be estimated once for a given camera (unless the camera has a variable focal length), the extrinsic parameters must be recomputed whenever cameras are moved or reoriented (on purpose or accidentally). In this paper, we assume that the intrinsic parameters are known, and we focus on extrinsic calibration.

Classical methods [1,2,3,4] require a calibration object with known world coordinates. They can be applied if there are sufficient point correspondences between 3D world points on the calibration object and corresponding image points. Calibration of a single camera can be done very efficiently with these methods, but calibration of a camera network is tedious and cumbersome to calibrate all of the cameras simultaneously, as it is often extremely difficult to make all of the points on the calibration object simultaneously visible in all views. Moreover, it involves the design and use of some highly accurate tailor-made calibration patterns, which are often difficult and expensive to manufacture.

Zhang [5] proposed a calibration algorithm that uses a planar grid pattern as the calibration object. This method is mainly for intrinsic calibration. It can also do the extrinsic calibration for a stereo case with a short baseline as soon as the planar pattern can be seen simultaneously by both cameras. Patterns required for this method [5] are easy and inexpensive to manufacture, which makes it flexible. However, for extrinsic calibration of a camera network or stereo case with a wide baseline, this method also encounters the problem of simultaneous visibility.

In order to avoid the problem of occluded features of a calibration object, many methods [6,7,8] have been proposed to create a virtual calibration object by simply moving a detectable point through the working volume. Svoboda *et al.* [6] proposed an approach for intrinsic and extrinsic calibration of camera networks using a moving laser pointer emitting a bright spot. A large number of correspondences of the virtual calibration object can be detected by waving the bright spot in front of the cameras. Their method firstly estimates the projective depth using the method of Sturm *et al.* [9], which exploits epipolar geometry. Then, it computes projective structures via Rank 4 factorization and does Euclidean stratification based on the concept of the absolute conic [10,11]. Finally, the bundle adjustment [12] is applied to refine the calibration. As the laser pointer needs to be moved many times, it is not easy to avoid occlusions in all camera views simultaneously. What is more, their method requires at least eight training samples (non-coplanar) and three cameras, which makes it not applicable to the calibration of a camera pair. Moreover, they cannot provide scale information without metric measurements.

Shen *et al.* [13] present an algorithm that simultaneously and automatically calibrates the extrinsic parameters across multiple color and depth cameras across the network. Rather than using the standard checkerboard, they used a sphere as a calibration object to identify the correspondences across different views. Guan *et al.* [14] also proposed a novel extrinsic calibration method for camera networks using a sphere as the calibration object. First of all, they propose an easy and accurate method to estimate the 3D positions of the sphere center w.r.t. the local camera coordinate system. Then, they used orthogonal Procrustes analysis to pairwise estimate the initial camera relative extrinsic parameters based on the aforementioned estimation of 3D positions. Finally, an optimization routine was applied to jointly refine the extrinsic parameters for all cameras.

All aforementioned calibration methods need specially designed calibration patterns and need to do the offline pre-calibration and re-calibration before or during the working phase of camera networks. For VSS, which mainly deals with the analysis of humans, pedestrians are predestined to be used as the calibration object for self-calibrating camera networks.

There are some prerequisites for our method. First, we assume that all cameras are synchronized and intrinsically calibrated. We also assume that all cameras share a common volume. In theory, only pairwise overlap is required, as we do calibration in a pairwise fashion. The region of overlap should be significant in order to get distinct calibration samples. By assuming the known height of the person, we can provide scale information. In order to not confuse the head and feet of the pedestrian between two camera views, we assume that the cameras may not be turned upside down. If we want to find extrinsic parameter of all cameras w.r.t a predefined world coordinate system, we need some ground truth measurements. We also assume that the posture of the person remains fixed (the lines from head to feet need to be parallel) while walking. Detecting image positions of the head and feet would be easier if the scene, except the pedestrians, remains static when pedestrians are walking. However, if the scene is not static, we can apply an adaptive background/foreground segmentation algorithm.

Firstly, we use and evaluate two methods to extract image positions of the feet and head using a classical foreground background segmentation technique and line fitting and ellipse fitting methods. Then, a novel method is proposed to estimate the 3D positions of the head and feet w.r.t. the local camera coordinate system assuming that the posture of the person remains fixed while walking.

As we can estimate the 3D position of the head and feet w.r.t. the local camera coordinate system, we propose to use the method of Arun *et al.* [15], which is based on orthogonal Procrustes analysis to obtain initial estimates of pairwise extrinsic calibration matrices. Arun *et al.* [15] proved that extrinsic parameters can still be uniquely found even when all corresponding 3D points (head and feet) are coplanar, which happens when the person walks along a straight line. We also combine RANSAC with the orthogonal Procrustes approach to deal with outliers in the observed data, which makes our method more robust against outliers. We then apply an optimization method developed by Bouguet [16], to jointly refine the extrinsic parameters for all cameras. The optimization step minimizes the total reprojection error (see Section 6) of calibration samples over all of the extrinsic parameters.

The first contribution of the paper is that our method is robust to the uncertainty of estimating persons’ head and feet positions in the image. We will show in Section 7.1.1 that the proposed method is more robust to the precise detection of head and feet positions compared to the method of Hödlmoser *et al.* [17], which does extrinsic calibration based on classical epipolar geometry [18]. The method from [17] first estimates the essential matrix [18] using image positions of head and feet as corresponding points. Then, it computes the extrinsic parameters by decomposing the essential matrix [10]. The method requires at least eight non-coplanar points, which means the method cannot work when the person walks along a straight line. Moreover, it requires multiple triangulation [19] steps to find the unique extrinsic parameters and scale information, which makes the method more prone to erroneous correspondences between heads (or feet) in different camera views.

A second contribution is that we compare our algorithm with the calibration method of Hödlmoser *et al.* [17] using a different number of locations and show that our method outperforms this method from literature. In Section 7.1.2, we will show that the projection, the reprojection and the triangulation errors of our method are always smaller than the errors in the method of Hödlmoser *et al.* We will also show that our method always produces more accurate and stable results when the person just stands at a few positions (fewer than eight). This case is quite common, especially if the common view of all cameras is limited. An extra benefit of our method is that it still provides valid extrinsic parameter estimates when the observed person just stands at two different positions. Methods based on the essential matrix cannot handle this case.

The third and most important contribution is that our method can be applied to the case where the pedestrian walks or even runs along a straight line. This case is quite common, especially if only a small part of the pedestrian’s path is within view of the camera. In Section 7.2, we will show that our method still provides accurate calibration in this case, whereas classical methods cannot. Specifically, the method based on the essential matrix cannot handle this case because of the co-planar location of all head and feet positions.

The final contribution is that our method can be easily extended to the case where multiple pedestrians are in the scene. In Section 7.3, we will show that our method still provide accurate calibration when there are three pedestrians in the scene.

The remainder of this paper is organized as follows. Section 2 gives a survey of works in the literature for extrinsic calibration. Section 3 provides details about the camera model and the architecture of the proposed calibration method. Section 4 explains how to estimate 3D positions of feet and head w.r.t. the camera coordinate system. Section 5 discusses the actual extrinsic calibration procedure based on the aforementioned 3D positions estimation. In Section 6, we explain five criteria for the accuracy evaluation of camera calibration techniques. Section 7 shows the experimental results. Finally, Section 8 concludes the paper.

## 2. Related Work

In recent years, extrinsic calibration of camera networks has been well studied both in the photogrammetric and the computer vision community. We discuss the related work on extrinsic calibration by observing pedestrians.

Many methods [20,21,22,23,24] have been proposed using pedestrians as the calibration object based on calibration from vanishing points [25]. Lv *et al.* [20] firstly recover the vertical vanishing point by observing walking people. Then, they estimate the horizon line by observing the person’s motion in different directions (the locations of the person are not all on a straight line). They can estimate both intrinsic and extrinsic parameters, but they need to specify two auxiliary lines that are parallel to the ground plane and mutually orthogonal to each other, which makes it not that feasible in practice. Liu *et al.* [23] proposed an algorithm that was capable of calibrating the camera using the known height distribution of pedestrians; it also uses ellipse fitting to detect persons’ heads and feet in the image as proposed in the method of Lv *et al.* [20]. However, it is mainly for intrinsic calibration and only applies to a single view. This work was then improved and extended to camera network calibration in [24]. First, each individual camera was roughly calibrated into its local world coordinate system based on the analysis of the relative 3D pedestrian height distribution. Then, all local world coordinate systems were iteratively registered with respect to a shared global world coordinate system by incorporating robust matching with a partial direct linear transform. This method [24] was also adopted by the method [26] for camera calibration and person re-identification. The method proposed in [27] not only uses pedestrians, but also a “Manhattan” scene with orthogonal structures, such as buildings and road lines. It uses similar methods as proposed by Liu *et al.* [23], and it is also only applicable to a single view. Schels *et al.* [28] also proposed a method similar to [23]. A drawback of the aforementioned methods is that they rely on estimating vanishing points, which is usually the bottleneck of approaches, because it is extremely sensitive to noise, as reported by Micuisik *et al.* [29]. Moreover, the common assumption made by these methods [20,21,22,23,24,27,28] is that they treat people as vertical sticks walking along a planar surface, which makes these methods only applicable to the case where people walk on a smooth horizontal surface. In contrast, our method does not require that the person walks on a plane surface (e.g., we allow walking on steps and stairs), as long as the posture of the person remains fixed (the lines from head to feet need to be parallel).

Possegger *et al.* [30] proposed an unsupervised extrinsic self-calibration method for a network of static cameras and pan–tilt–zoom (PTZ) cameras solely based on correspondences between tracks of a walking human. First, they tracked a walking human throughout the scene and computed the foot and head locations for every camera in the network. Next, they removed outliers in the detected foot and head measurements to obtain clean measurements for the calibration step. Finally, they performed a modified bundle adjustment to estimate the extrinsic parameters of the cameras. As they needed to solve the non-linear least squares optimization problem, their method tends to get stuck in local optima, unless it is initialized with good estimates (which was not given in their method).

For extrinsic calibration of cameras with known intrinsic parameters, epipolar geometry also plays an import role. Many methods have been proposed for calibration using epipolar geometry [6,7,8]. Longuet-Higgins [18] showed how an essential matrix relating a pair of calibrated views can be estimated from eight or more point correspondences by solving a linear equation and also how the essential matrix can be decomposed to give relative camera orientation and position. Hödlmoser *et al.* [17] use the head and feet of the person as the corresponding points to estimate the essential matrix. Then, they decompose the essential matrix to obtain the camera rotation and translation parameters. However, decomposing the essential matrix, multiple triangulations are needed for the chirality check [31,32], which makes the method more prone to erroneous correspondences between heads (or feet) in different camera views. Moreover, the method using the essential matrix will fail when pedestrians walk along a straight line, which occurs quite often in practice. In that case, all head and feet positions lie in a plane, which is a degenerate case for estimating the essential matrix [10]. Only the homography matrix between two views can be obtained, but it is not possible to get unique extrinsic parameters by decomposing the homography matrix [33,34].

Instead of using epipolar geometry and image positions directly, we propose an easy and accurate method to estimate 3D positions of feet and head w.r.t. the camera coordinate system. Then, we estimate extrinsic parameters by computing the 3D rigid body transformation that optimally aligns two sets of points for which the correspondence is known [35]. Our method still works when pedestrians walk along a straight line as we can estimate the 3D positions w.r.t. a single camera coordinate system. It is proven in the method of Arun *et al.* [15] that extrinsic parameters can still be uniquely found even when all corresponding 3D points are coplanar.

Tardif *et al.* [36] proposed to use straight line structures from the scene to estimate the radial distortion in the camera image. Images of line patterns were used to formulate linear constraints on the distortion function parameters. This method was extended by Houben [37] for estimating distortion parameters, as the first step of their self-intrinsic calibration method. The common constraint used in those two methods [36,37] is that three collinear points in the world should still be collinear in the rectified image; which is a bit similar, but different from the constraint we use when estimating the 3D positions of the head and feet w.r.t. the local camera coordinate system. We explore the fact that the three vectors, 3D head position, 3D feet position and the unit vector of the person, are always coplanar. We also assume that the unit vector representing the person remains the same while walking, from which we can estimate the unit vector of the person w.r.t. a single camera coordinate system. The details will be introduced in Section 4.2.

## 3. Preliminaries

### 3.1. Camera Model

Extrinsic parameters are expressed with respect to a reference coordinate system, which is also called the world coordinate system. In this system, a point in 3D space is denoted as $r^{\left(w\right)}={\left(X_{w},Y_{w},Z_{w}\right)}^{T}$, where the superscript *T* denotes a matrix transposition. We also associate a distinct camera coordinate system with each camera. In the system for camera *k*, a 3D point is denoted as $r^{\left(k\right)}={\left(X^{\left(k\right)},Y^{\left(k\right)},Z^{\left(k\right)}\right)}^{T}$. If we want to estimate the extrinsic parameters of all cameras w.r.t. a predefined world coordinate system, we need to measure coordinates of some 3D points in this coordinate system. In order to avoid manually measuring 3D positions, we choose the coordinate system of the first camera as the world coordinate system: $r^{\left(w\right)}=r^{\left(1\right)}$, which is also the assumption made in the classical self-calibration method [11]. The camera coordinates $r^{\left(k\right)}$ are related to the world coordinates $r^{\left(w\right)}$ by $r^{\left(k\right)}=R^{\left(k\right)}r^{\left(w\right)}+c^{\left(k\right)}$, where $c^{\left(k\right)}$ are the coordinates of the origin of the global world coordinate system within the local coordinate system of camera *k* and $R^{\left(k\right)}$ is a 3×3 rotation matrix. The purpose of this paper is to find $c^{\left(k\right)}$ and $R^{\left(k\right)}$ for each camera.

Intrinsic parameters define the imaging geometry of the camera. Since we deal with only one camera for intrinsic parameters, we will drop the superscripts *k*. In the camera coordinate system, a point is denoted as $r={\left(X,Y,Z\right)}^{T}$. We assume that the camera is modeled by the usual pinhole, where the coordinate system has its origin in the optical center of the camera, and the *Z* axis (optical axis) is perpendicular to the image plane. Therefore, the image plane is given by Z=f, where *f* is the focal length of the camera in physical units (e.g., centimeter). Moreover, in each camera image, the projection of a 3D point can be characterized by its normalized image coordinates x=(x,y), which have the same physical units as the camera coordinates r. Alternatively, a projected point can also be identified by its integer pixel coordinates $u={\left(u,v\right)}^{T}$, *i.e.*, a column and row number in the image. We use $\tilde{x}$ (also known as homogeneous coordinates) to denote the augmented vector by adding one as the last element: $\tilde{x}={\left(x,y,1\right)}^{T},\tilde{u}={\left(u,v,1\right)}^{T}$. Therefore, there are three specific coordinate systems associated with each camera observation of a point. The first set of coordinates r is expressed in physical units and indicates the 3D position of a point relative to the camera. The second set and third sets of coordinates are the normalized image coordinates x expressed in physical units and the coordinates u, expressed in pixels, respectively.

Assuming a zero-skew camera and f=1, the pixel and normalized image coordinates are related by:

(1) $$\tilde{u}=A\tilde{x}$$

where *A* is the camera intrinsic matrix, which is given by:

(2) $$\begin{array}{c} A=\left[\begin{array}{ccc} f_{x} & 0 & u_{0}\\ 0 & f_{y} & v_{0}\\ 0 & 0 & 1 \end{array}\right] \end{array}$$

with $\left(u_{0},v_{0}\right)$ the coordinates of the principal point, $f_{x},f_{y}$ the scale factors in the image *u* and *v* axes. Moreover, $f_{x}=f/s_{x}=1/s_{x},f_{y}=f/s_{y}=1/s_{x}$, in which $s_{x},s_{y}$ are the pixel dimensions in physical units. The 3D coordinates r and the normalized image coordinates $\tilde{x}$ are related as:

(3) $$r=Z\tilde{x}$$

Combining Equations (1) and (3), we get:

(4) $$r=ZA^{-1}\tilde{u}$$

Thus, we can estimate the 3D position of a point w.r.t. the camera coordinate system if we know *Z* (difficult), $\tilde{u}$ (to be extracted from the image) and *A* (known after intrinsic calibration).

### 3.2. Notations and Architecture

In this paper, we denote by $d_{3}\left(a_{3},b_{3}\right)$ the Euclidean distance between two 3D vectors $a_{3}$ and $b_{3}$. We also denote by $d_{2}\left(a_{2},b_{2}\right)$ the Euclidean distance between two 2D vectors $a_{2}$ and $b_{2}$.

Since the number of variables employed in this paper is high, we provide a table to define the variables, as shown in Table 1.

In Section 4, we will show how to estimate 3D positions of the head and feet based on the image of a walking person.

**Figure 1:**
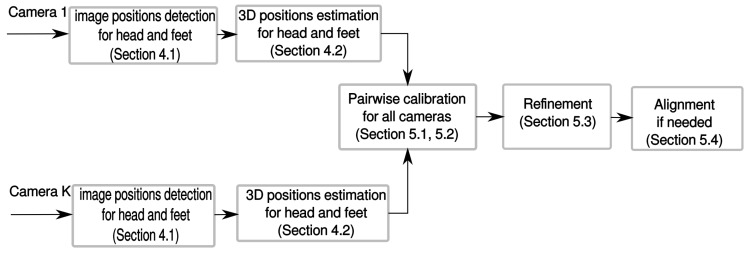
Architecture of the proposed calibration method.

In Section 5, we will provide the actual extrinsic calibration procedure based on the aforementioned 3D positions estimation. Figure 1 depicts the procedures of the proposed calibration method.

## 4. 3D Head and Feet Positions in Local Camera Coordinates

### 4.1. Extract Image Positions of Head and Feet

To determine the image positions of the head and feet of a walking person, we propose to detect the person’s silhouette in the first step. Since we are not mainly focusing on solving the problem of background subtraction and tracking, we just apply a well-known background subtraction method [38] to obtain a rough foreground blob of the pedestrian.

As there is uncertainty about estimating persons’ head and feet positions in the image, we propose and evaluate two different, but commonly-used methods to obtain the image coordinates $u_{h}$ and $u_{f}$, the person’s head and feet, respectively, from the foreground blob of the pedestrian.

1. Ellipse fitting: We fit an ellipse to the blob of the person. The end points of the major axis of the ellipse are then taken as $u_{h}$ and $u_{f}$.
2. Bounding box and line fitting: We fit a line and a bounding box to a person’s blob. The intersections between the line and the bounding box of the blob are taken as $u_{h}$ and $u_{f}$.

**Figure 2:**
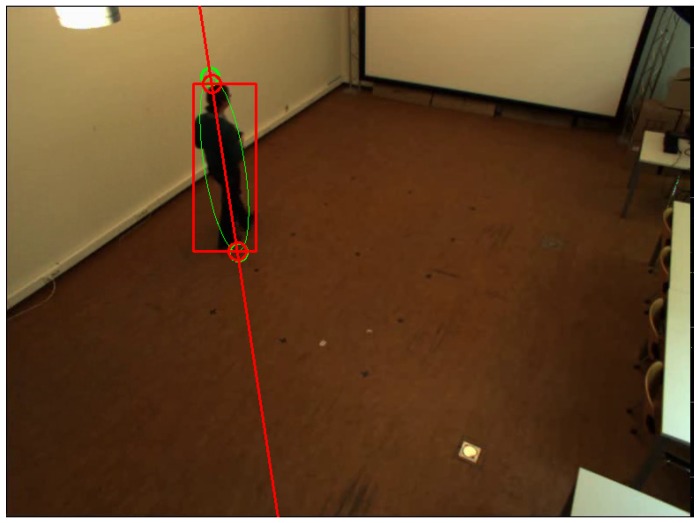
Head and feet detection in an image. Two green circles represent detected positions using ellipse fitting. Two red circles represent detected positions using bounding box fitting and line fitting.

Figure 2 shows the detected head and feet positions using the two aforementioned methods.

### 4.2. Estimate 3D Positions Based on Image Positions

**Figure 3:**
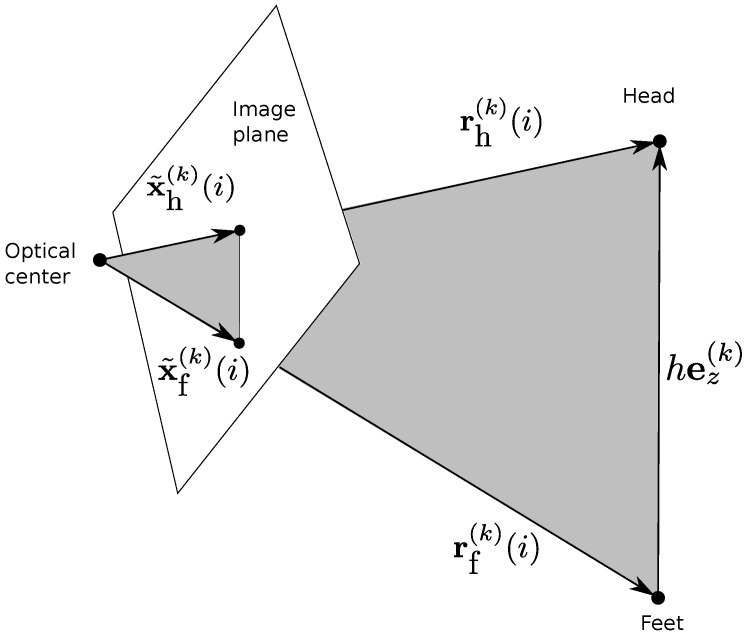
Co-planarity of $r_{h}^{\left(k\right)}\left(i\right)$, $r_{f}^{\left(k\right)}\left(i\right)$ and $he_{z}^{\left(k\right)}$. $he_{z}^{\left(k\right)}$ remains the same while walking.

Suppose a person moves between *N* different positions while keeping a fixed posture. Suppose that all cameras see both the feet and the head of the person. At each position, we first calculate ${\tilde{u}}_{f}^{\left(k\right)}\left(i\right)$ and ${\tilde{u}}_{h}^{\left(k\right)}\left(i\right)$ using the technique described in Section 4.1, with i=1,2…N the index of the relevant position and k=1,2…K the index of the relevant camera. Let ${\tilde{x}}_{f}^{\left(k\right)}\left(i\right)$ and ${\tilde{x}}_{h}^{\left(k\right)}\left(i\right)$ be the normalized image coordinates (x,y,1) of the feet and the head. Furthermore, let $Z_{f}^{\left(k\right)}\left(i\right)$ and $Z_{h}^{\left(k\right)}\left(i\right)$ be the corresponding unknown $Z^{\left(k\right)}$ coordinates. Finally, assume that the person is walking upright and has height *h*, and let $r_{f}^{\left(k\right)}\left(i\right)$ and $r_{h}^{\left(k\right)}\left(i\right)$ be the 3D camera coordinates of the head and feet. By defining $he_{z}^{\left(k\right)}$ the vector of the person’s center line within camera *k*, we have $r_{h}^{\left(k\right)}\left(i\right)=r_{f}^{\left(k\right)}\left(i\right)+he_{z}^{\left(k\right)}$, since the vectors of $r_{h}^{\left(k\right)}\left(i\right)$, $r_{f}^{\left(k\right)}\left(i\right)$ and $he_{z}^{\left(k\right)}$ compose a triangle. Figure 3 shows the triangle composed by these three vectors. With $r_{h}^{\left(k\right)}\left(i\right)=Z_{h}^{\left(k\right)}\left(i\right){\tilde{x}}_{h}^{\left(k\right)}\left(i\right)$ and $r_{f}^{\left(k\right)}\left(i\right)=Z_{f}^{\left(k\right)}\left(i\right){\tilde{x}}_{f}^{\left(k\right)}\left(i\right)$, we have:

(5) $$\begin{array}{ccc} Z_{h}^{\left(k\right)}\left(i\right){\tilde{x}}_{h}^{\left(k\right)}\left(i\right)-Z_{f}^{\left(k\right)}\left(i\right){\tilde{x}}_{f}^{\left(k\right)}\left(i\right) & = & he_{z}^{\left(k\right)}\mathrm{with}e_{z}^{\left(k\right)}\overset{▵}{=}R^{\left(k\right)}e_{z} \end{array}$$

in which $e_{z}$ is the unit vector of the person in the world coordinates system.

Now, let us define $m^{\left(k\right)}\left(i\right)={\tilde{x}}_{f}^{\left(k\right)}\left(i\right)\times {\tilde{x}}_{h}^{\left(k\right)}\left(i\right)$ with × representing the cross product, which is a vector orthogonal to ${\tilde{x}}_{f}^{\left(k\right)}\left(i\right)$ and ${\tilde{x}}_{h}^{\left(k\right)}\left(i\right)$. It is also the normal vector of the plane spanned by two vectors ${\tilde{x}}_{f}^{\left(k\right)}\left(i\right)$ and ${\tilde{x}}_{h}^{\left(k\right)}\left(i\right)$. Since $he_{z}^{\left(k\right)}$ is also on that plane, we have ${\left(m^{\left(k\right)}\left(i\right)\right)}^{T}he_{z}^{\left(k\right)}=0$; cancelling *h* leads to:

(6) $${\left(m^{\left(k\right)}\left(i\right)\right)}^{T}e_{z}^{\left(k\right)}=0$$

Let $M^{\left(k\right)}$ be the matrix with rows ${\left(m^{\left(k\right)}\left(i\right)\right)}^{T}$, we have:

(7) $$M^{\left(k\right)}e_{z}^{\left(k\right)}=0$$

Therefore, $e_{z}^{\left(k\right)}$ must be in the null space of the matrix $M^{\left(k\right)}$. Hence, $e_{z}^{\left(k\right)}$ is determined by SVD of $M^{\left(k\right)}$.

Once $e_{z}^{\left(k\right)}$ is known and with *K* cameras, Equation (5) becomes a system of 3K equations with 2K+1 unknowns: $Z_{f}^{\left(k\right)}\left(i\right)$, $Z_{h}^{\left(k\right)}\left(i\right)$ and *h*. This system of equations is solved in the least squares sense for the variables $Z_{f}^{\left(k\right)}\left(i\right)$ and $Z_{h}^{\left(k\right)}\left(i\right)$, which are thus determined up to a constant factor *h*. In the following, it makes a difference about the scale information of the system if we treat *h* as an unknown or not. If we assume a different *h* than the correct one, the result will be equivalent to a re-scaling of the global coordinate system. Once $Z_{f}^{\left(k\right)}\left(i\right)$ and $Z_{h}^{\left(k\right)}\left(i\right)$ have been found, we obtain the 3D positions of the feet and head w.r.t. the local camera coordinate system using $r_{h}^{\left(k\right)}\left(i\right)=Z_{h}^{\left(k\right)}\left(i\right){\tilde{x}}_{h}^{\left(k\right)}\left(i\right)$ and $r_{f}^{\left(k\right)}\left(i\right)=Z_{f}^{\left(k\right)}\left(i\right){\tilde{x}}_{f}^{\left(k\right)}\left(i\right)$.

The aforementioned technique estimates the 3D positions of head and feet w.r.t the local camera coordinate system, by analyzing multiple observations (at least 2) of a single person. This requirement is equivalent to the case where a single image of multiple person exists. In this case, we assume a average height of multiple persons, and also assume that the center lines of different persons are parallel. Then we can use the same aforementioned technique to estimate 3D positions of the head and feet for different persons in a single image.

## 5. Multi-Camera Calibration

From the equations in Section 4, we calculate the 3D camera coordinates of the head and feet of a person. Then, we compute the overall coordinate transform, which optimally aligns two sets of 3D points (head and feet) for which the correspondence is known.

Many methods have been proposed to estimate the rigid body transformation between two sets of 3D points. Eggert *et al.* [35] compared four popular and efficient algorithms [15,39,40,41] and found that the method of Arun *et al.* [15] provides the best overall accuracy and stability. We therefore use that method, which is based on orthogonal Procrustes analysis [42].

### 5.1. Pairwise Calibration Based on Orthogonal Procrustes

As we select the coordinate frame of Camera 1 as the world coordinate frame, we have $r^{\left(k\right)}=R^{\left(k\right)}r^{\left(1\right)}+c^{\left(k\right)}$. We first find the extrinsic parameters of all cameras w.r.t. the first camera using orthogonal Procrustes. Observing a person in *N* different locations provides 2N points (heads and feet) for each camera. The coordinates of these points in different cameras are related by: $r^{\left(k\right)}\left(i\right)=R^{\left(k\right)}r^{\left(1\right)}\left(i\right)+c^{\left(k\right)}$, with i=1,2…2N. In order to find $R^{\left(k\right)}$ and $c^{\left(k\right)}$ using orthogonal Procrustes, we need to decouple the translation and rotation. Since the centroid of a point cloud remains the same no matter from which camera view, we cancel translation by moving the origin from the camera center to the centroid of the point cloud. We therefore calculate the centroid of $r^{\left(1\right)}\left(i\right)$ and $r^{\left(k\right)}\left(i\right)$ using:

(8) $$\begin{array}{ccc} \bar{r^{\left(1\right)}}=\frac{1}{2N}\sum_{i=1}^{2N}r^{\left(1\right)}\left(i\right), & & \bar{r^{\left(k\right)}}=\frac{1}{2N}\sum_{i=1}^{2N}r^{\left(k\right)}\left(i\right) \end{array}$$

Let $H^{\left(1\right)}$ be the matrix with columns $r^{\left(1\right)}\left(i\right)-\bar{r^{\left(1\right)}}$, and $H^{\left(k\right)}$ be the matrix with columns $r^{\left(k\right)}\left(i\right)-\bar{r^{\left(k\right)}}$; then, we have $H^{\left(k\right)}=R^{\left(k\right)}H^{\left(1\right)}$ or, equivalently, ${H^{\left(k\right)}}^{T}={H^{\left(1\right)}}^{T}{R^{\left(k\right)}}^{T}.$ Using orthogonal Procrustes, we decompose $H^{\left(1\right)}{H^{\left(k\right)}}^{T}$ as $H^{\left(1\right)}{H^{\left(k\right)}}^{T}=U_{k}S_{k}V_{k}^{T}.$ As pointed out by Arun *et al.*, there will be three possibilities for the solution of $R^{\left(k\right)}$ from geometrical considerations.

1. The points $r^{\left(k\right)}\left(i\right)$ are not coplanar. In this case, the rotation matrix is uniquely found and calculated by $R^{\left(k\right)}=V_{k}U_{k}^{T}$.
2. The points $r^{\left(k\right)}\left(i\right)$ are coplanar, but not collinear. In this case, the rotation matrix is calculated using:
  (9) $$R^{\left(k\right)}=\left\{\begin{array}{cc} V_{k}U_{k}^{T} & \text{if}\;\det(V_{k}U_{k}^{T})=1\\ V_{k}^{{}^{\prime }}U_{k}^{T} & \text{if}\;\det(V_{k}U_{k}^{T})=-1 \end{array}\right.$$
  where $V_{k}^{{}^{\prime }}$ is obtained by changing the sign of the last column of matrix $V_{k}$.
3. The points $r^{\left(k\right)}\left(i\right)$ are collinear. In this case, $R^{\left(k\right)}$ cannot be uniquely found, which is the failure case of our method.

Once we obtain the rotation matrix $R^{\left(k\right)}$, the translation vector $c^{\left(k\right)}$ is then given by:

(10) $$\begin{array}{c} c^{\left(k\right)}=\bar{r^{\left(k\right)}}-R^{\left(k\right)}\bar{r^{\left(1\right)}} \end{array}$$

### 5.2. Robust Calibration Using RANSAC

The technique described in Section 5.1 can provide a good estimation of extrinsic parameters when there are no outliers in the estimated 3D points. However, in practice, the feet of the person sometimes are prone to occlusion, since they are close to the ground. In this case, the extracted 3D position of the feet in one camera (occluded) does not correspond well with its position in another camera (not occluded). Moreover, different cameras may see different points of the head or feet, as there are many detectable points on the head or feet, which will bring bad correspondence. Therefore, we propose to combine RANSAC [43] with the method proposed in Section 5.1, from which we deal with outliers in the dataset and make the estimation of extrinsic parameters robust.

The steps of the RANSAC scheme are as follows:

1. Select three pairs of 3D points randomly and compute extrinsic parameters using the method from Section 5.1.
2. Count the number of pairs agreeing with the extrinsic parameters (inliers). A pair $\left(r^{\left(1\right)}\left(i\right),r^{\left(k\right)}\left(i\right)\right)$ is considered to agree with the extrinsic parameters if for some threshold *ϵ*:
  (11) $$d_{3}\left(r^{\left(k\right)}\left(i\right),R^{\left(k\right)}r^{\left(1\right)}\left(i\right)+c^{\left(k\right)}\right)<\epsilon$$
3. Repeat Steps 1 and 2 until the number of inliers reaches a certain threshold.
4. Re-compute extrinsic parameters using all of the inliers based on the method from Section 5.1.

### 5.3. Refinement through Gradient Descent

Section 5.1 and Section 5.2 provide a quite good estimate of the extrinsic calibration parameters, but they are not jointly optimized, since we do pairwise calibration to relate each camera to the first camera. Additionally, the above solution is obtained through minimizing an algebraic distance, which does not take the property of the cameras’ projective geometry into account. To jointly refine the extrinsic parameters for all cameras, we apply an optimization method that was developed by Bouguet [16]. The optimization step minimizes the total reprojection error over all of the extrinsic parameters. The objective function is the mean-squared discrepancy between the observed positions of feet and head in the image and their image reprojections computed using the estimated extrinsic calibration matrices. It is optimized by an iterative gradient descent procedure.

### 5.4. Alignment with a World Coordinate System

The aforementioned calibration yields the extrinsic camera parameters in the coordinate frame of the first camera. In practical applications, it is often desirable to have all parameters in some user-specified world coordinate system. For example, for camera networks that are intended for indoor people tracking, we would like to have the Z=0 plane to coincide with the ground floor. For that, and only for that, we need ground truth measurements of at least three points (alignment samples) w.r.t. the user-specified world coordinate system.

We first estimate 3D positions (w.r.t. the coordinate system of the first camera) of these points based on the initial estimate extrinsic parameters and known intrinsic parameters using the triangulation method [19]. As the estimated 3D positions are up to a scale (the person’s height), we first need to estimate the scale of the camera network. For two alignment samples with positions $r^{\left(w\right)}\left(1\right)$ and $r^{\left(w\right)}\left(2\right)$ in the user-specified world coordinate system, we calculate the ground truth Euclidean distance between these two samples as $d_{1,2}^{\left(w\right)}=d_{3}\left(r^{\left(w\right)}\left(1\right),r^{\left(w\right)}\left(2\right)\right)$. After we obtain the extrinsic parameters of other cameras w.r.t. the coordinate system of the first camera, we obtain the 3D positions (w.r.t. the coordinate system of the first camera) of the same two samples using triangulation based on the corresponding 2D image coordinates and estimated extrinsic parameters. Then, we have $d_{1,2}^{\left(c\right)}=d_{3}\left(r^{\left(1\right)}\left(1\right),r^{\left(1\right)}\left(2\right)\right)$. This distance is computed in the camera coordinate system, but of course, it should equal the ground truth distance up to a scale in the absence of calibration and image processing errors. We calculate the scale factor using $\lambda_{1,2}=d_{1,2}^{\left(w\right)}/d_{1,2}^{\left(c\right)}$. Since we have multiple alignment samples, we calculate the mean value of the scale factor over all combinations of any two samples.

After obtaining the scale factor, we apply the scale information to all estimated 3D positions and use the Procrustes approach of Section 5.1 to compute the transform between the user-specified coordinate system and Camera 1’s coordinate system.

## 6. Performance Measures

### 6.1. Measures without Ground Truth

We firstly propose to evaluate the proposed calibration method by how well we can measure the 3D world and how well we can project a 3D point to the image using the extrinsic parameters obtained through our calibration method. In the following, we assume that we have acquired *K* images of *n* markers (points in the observed space), which are used as test samples to evaluate the calibration accuracy. We also assume that the ground truth coordinates (w.r.t. the user-specified world coordinate system) of these test samples are known. In practice, we measure them using a tape measure.

1. $\delta r^{\left(w\right)}$. The triangulation error is a measure of how the calibration matrices influence the accuracy of multi-camera triangulation. Let $r_{i}^{\left(w\right)}$ be the ground truth position of the *i*-th test sample and ${\hat{r}}_{i}^{\left(w\right)}$ its position estimated using the triangulation method of [19], which takes as input the estimated extrinsic parameters and image positions of the *i*-th test markers. This is also the classical method for 3D reconstruction: it represents how well we can measure the 3D world with estimated extrinsic parameters. The discrepancy between real and estimated positions is compared. The error is expressed in physical units (e.g., centimeter) and is defined as:
  (12) $$\delta r^{\left(w\right)}=\frac{1}{n}\sum_{i=1}^{n}d_{3}\left(r_{i}^{\left(w\right)},{\hat{r}}_{i}^{\left(w\right)}\right)$$
  The main limitation of this measure is that it depends on the number of cameras used in the triangulation. With increasing number of cameras, the influence of errors in calibration matrices will tend to decrease. The main advantage of the measure is that is indicative of the accuracy that can be achieved in many real-world applications.
2. $\delta u^{p}$. The projection error is a measure of how the calibration matrices influence the accuracy of projections of 3D points on image planes. Let $u_{ik}$ be the observed pixel coordinates of the *i*-th sample in the *k*-th camera’s image, while ${\hat{u}}_{ik}^{p}$ is the estimated position through projection. Accuracy is obtained by measuring the discrepancy between the real 2D points (obtained from image segmentation) and the estimated ones (obtained by using the camera model). The error is expressed in pixels and is defined as:
  (13) $$\delta u^{p}=\frac{1}{nK}\sum_{i=1}^{n}\sum_{k=1}^{K}d_{2}\left({\hat{u}}_{ik}^{p},u_{ik}\right)$$
  The main limitation of this measure is that it not only depends on the extrinsic and intrinsic parameters, but also depends on the measurement from image segmentation. Therefore it is not an absolute measurement of the calibration accuracy. However, since the same image feature points are used to compare different calibration methods, $\delta u^{p}$ is another good quality measurement for the extrinsic calibration.
3. $\delta u^{r}$. The reprojection error is used to quantify how closely we can recreate the point’s true projection $u_{ik}$ with an estimate of a 3D point ${\hat{r}}_{i}^{\left(w\right)}$. Different from the projection error, the 3D points are firstly obtained from triangulation based on estimated extrinsic parameters and image points. Then, image feature points are projected from these 3D points. The discrepancy between the real 2D points $u_{ik}$ (obtained from image segmentation) and the estimated ones ${\hat{u}}_{ik}^{r}$ (obtained through reprojection) is computed. The error is expressed in pixels and is defined as:
  (14) $$\delta u^{r}=\frac{1}{nK}\sum_{i=1}^{n}\sum_{k=1}^{K}d_{2}\left({\hat{u}}_{ik}^{r},u_{ik}\right)$$
  The main limitation of this measure is that it not only depends on the extrinsic and intrinsic parameters, but also depends on the triangulation method and the measurement from image segmentation. Therefore, it is not an absolute measurement of the calibration accuracy. However, since the same image feature points are used to compare different calibration methods, $\delta u^{r}$ is another good quality measurement for the extrinsic calibration.

### 6.2. Measures with Ground Truth

Alternatively, we can get ground truth for extrinsic parameters by doing calibration using classical methods [1] with sufficient point correspondences between 3D world points (w.r.t. a predefined world coordinate system) and corresponding image points. Since extrinsic parameters are composed of rotation and translation parameters, we provide two measures to evaluate them separately.

1. δc. The relative translation error is used to quantify how closely we can estimate the distance between the camera center and the origin of the world coordinate system. We get $c^{\left(k\right)}$ for the *k*-th camera from classical methods [1]. We also estimate translation ${\hat{c}}^{\left(k\right)}$ with the proposed calibration method. Cameras are usually mounted high above the ground plane for getting a good view of the scene and increasing the viewing area. The origin of the world coordinate system mostly lies on the ground plane. Therefore, the distance between a camera center and the world origin is usually large (at least 200 cm). Thus, we propose to calculate relative translation error by:
  (15) $$\delta c=\frac{1}{K}\sum_{k=1}^{K}\frac{d_{3}\left(c^{\left(k\right)},{\hat{c}}^{\left(k\right)}\right)}{d_{3}\left(c^{\left(k\right)},0_{3}\right)}$$
  where $0_{3}$ is a 3D zero vector.
2. δθ. The rotation error is used to quantify how accurately we can estimate the orientation of all cameras. We get three ground truth angles ($\theta_{X}^{\left(k\right)},\theta_{Y}^{\left(k\right)},\theta_{Z}^{\left(k\right)}$) for the *k*-th camera From classical methods [1]. We also estimate the three angles (${\hat{\theta }}_{X}^{\left(k\right)},{\hat{\theta }}_{Y}^{\left(k\right)},{\hat{\theta }}_{Z}^{\left(k\right)}$) with the proposed calibration method. Then, we calculate rotation error by:
  (16) $$\delta \theta =\frac{1}{K}\sum_{k=1}^{K}\frac{\sqrt{{\left(\theta_{X}^{\left(k\right)}-{\hat{\theta }}_{X}^{\left(k\right)}\right)}^{2}}+\sqrt{{\left(\theta_{Y}^{\left(k\right)}-{\hat{\theta }}_{Y}^{\left(k\right)}\right)}^{2}}+\sqrt{{\left(\theta_{Z}^{\left(k\right)}-{\hat{\theta }}_{Z}^{\left(k\right)}\right)}^{2}}}{3}$$

## 7. Experiments and Results

### 7.1. Calibration when the Person Does not Walk along a Straight Line

For evaluation, we first calibrated a multi-camera tracking system composed of four side view cameras. The cameras were mounted at a height of about 3 m in each corner of a room (8.6 m by 4.8 m) and have a resolution of 780 by 580 pixel. These cameras were intrinsically calibrated using the method of Zhang [5]. We compare our method to the calibration method of Hödlmoser *et al.* [17], which exploits epipolar geometry [18]. For the purpose of comparison, both methods use the same intrinsic parameters. We used RANSAC-based algorithm to estimate the essential matrix, when we implemented the method of of Hödlmoser *et al.* [17]. We also obtained the ground truth for extrinsic parameters using classical method [1] implemented by Bouguet [16].

Some of the performance criteria in Section 6 require ground truth expressed in the user-specified world coordinate system. For this purpose, we measured the positions of four markers using a tape measure; those four markers are not on the same plane. These data are then used to convert the global camera coordinate system to a user-specified one with the method described in Section 5.4. To test the accuracy of calibration, we also captured 18 more test samples. In this case, the markers were placed in different positions than for the alignment samples.

**Figure 4:**
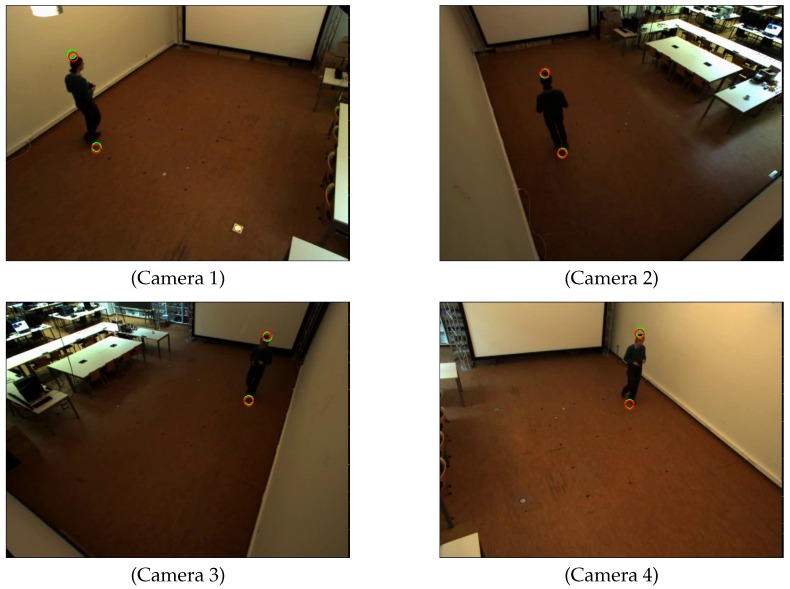
Detected feet and head positions for each camera. Green circles represent detected positions using ellipse fitting. Red circles represent detected positions using bounding box fitting and line fitting.

**Figure 5:**
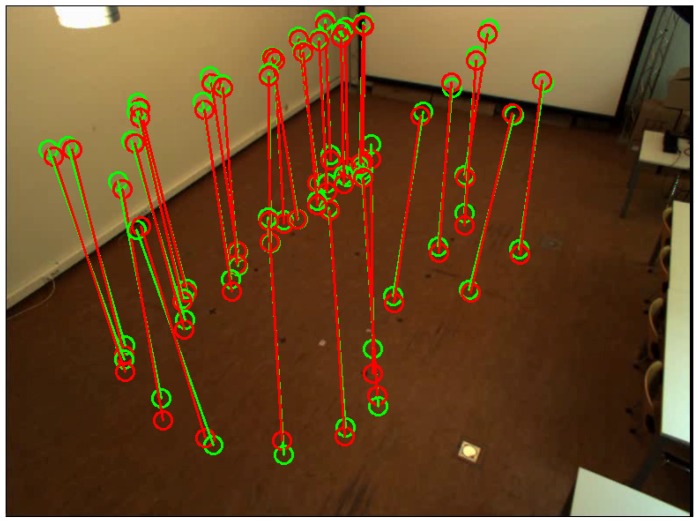
Distribution of the person’s positions for Camera Network 1. Green circles represent detected positions using ellipse fitting. Red circles represent detected positions using bounding box fitting and line fitting.

Figure 4 shows the detected head and feet positions of the person in each scene. We detected the head and feet positions when the person stood at 48 locations in the common view of all cameras. Figure 5 shows the distributions of the person’s positions. We can see that the detected positions of head and feet using the proposed two methods are close to each other for most of the person positions.

#### 7.1.1. Comparison Using Different Feet and Head Detections

In Section 4.1, we proposed two methods to detect head and feet positions of a person in an image. We have two sets of head and feet positions. To assess the robustness to the precise detection of head and feet positions, we use all two sets of feet and head positions to do the calibration. We repeat the calibration procedure 100 times; each time, we randomly select 8 locations from the available ones (48 locations of the person in total). Each location yields two calibration samples (head and feet).

Table 2 shows the comparison between our method and the method that explores the essential matrix in terms of five measures described in Section 6. As can be seen from Table 2, our method with refinement always produces the most stable and accurate calibration compared to the method of Hödlmoser *et al.* [17] (both with and without refinement) for the comparison, in terms of all five measures.

#### 7.1.2. Comparison Using Different Numbers of Locations

In this section, we evaluate both methods using different observation numbers of the person. We firstly did the comparison with fewer observations, which is important when the common view of all cameras is limited.

In order to show that our method can work accurately with fewer observations than other methods, we run the calibration procedure with *N* locations (1<N<8). For each value of *N*, repeat the calibration 1000 times using randomly-selected positions. We use detected head and feet positions from ellipse fitting, as it is natural to model the person as an ellipse. We count the times (success percentage) when the method provides a successful initial estimation of extrinsic parameters, in which “successful” means that the triangulation error is below 15 cm.

Estimating the essential matrix using a linear method (*i.e.*, DLT [10]) requires N≥4, *i.e.*, that method cannot work with fewer than four distinct locations. Table 3 shows that both methods provide better results with increasing *N*, *i.e.*, with more calibration data. We can also observe that the method of the essential matrix in reality does not produce good results for N=4: in only 35.3% of the cases, the results are good for N=4. The reason is that the method becomes numerically unstable when the locations are almost co-planar. The risk of this happening is large when N=4. Another reason is that the method could not find the correct extrinsic parameters by decomposing the essential matrix, when there is large error in the estimated essential matrix. The risk of this happening is also large when N=4.

In contrast, our method requires N≥2 (the person just needs to be at two different places). With N=2, still, our method succeeds 63.3% of the time. The reason is that the method becomes numerically unstable when the locations are almost identical. The risk of this happening is large when N=2.

Different from the aforementioned experiment, which did the comparison by counting the numbers of successful initial estimations of extrinsic parameters, when only limited observations of the person can be available, we then evaluated both methods using the criteria defined in Section 6 with more observations. We repeat the calibration procedure 100 times for each *N* (N>8), for various sets of locations, randomly selected from the available ones.

**Figure 6:**
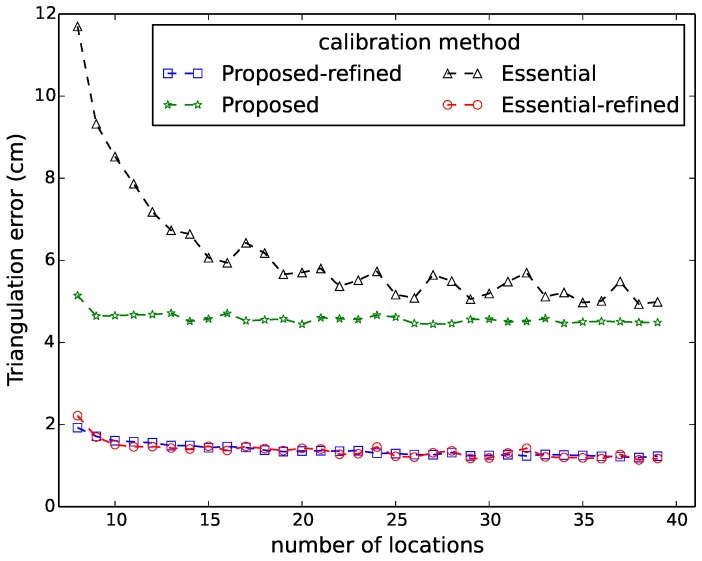
Triangulation error comparison between our method (proposed) and the method of Hödlmoser *et al.* [17] (essential) using different numbers of locations. Each location yields two calibration samples (head and feet).

**Figure 7:**
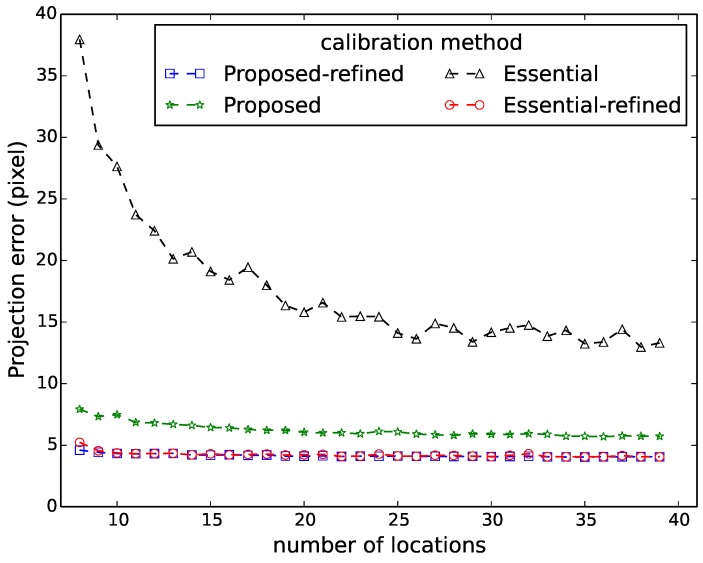
Projection error comparison between our method (proposed) and the method of Hödlmoser *et al.* [17] (essential) using different numbers of locations. Each location yields two calibration samples (head and feet).

**Figure 8:**
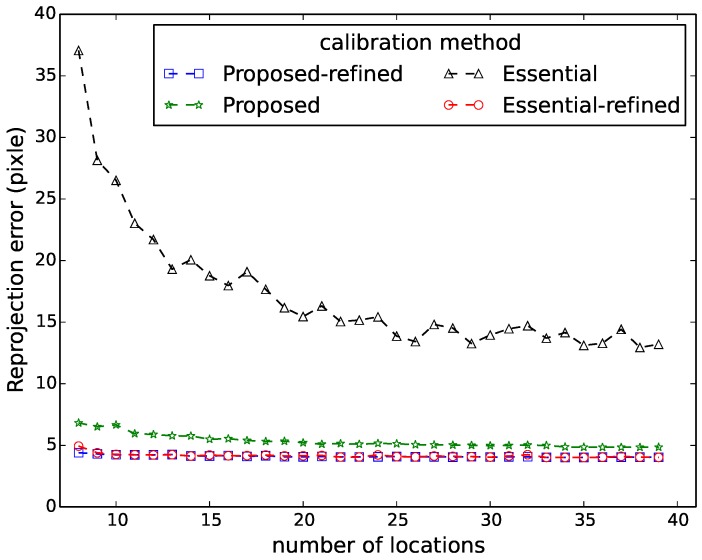
Reprojection error comparison between our method (proposed) and the method of Hödlmoser *et al.* [17] (essential) using different numbers of locations. Each location yields two calibration samples (head and feet).

**Figure 9:**
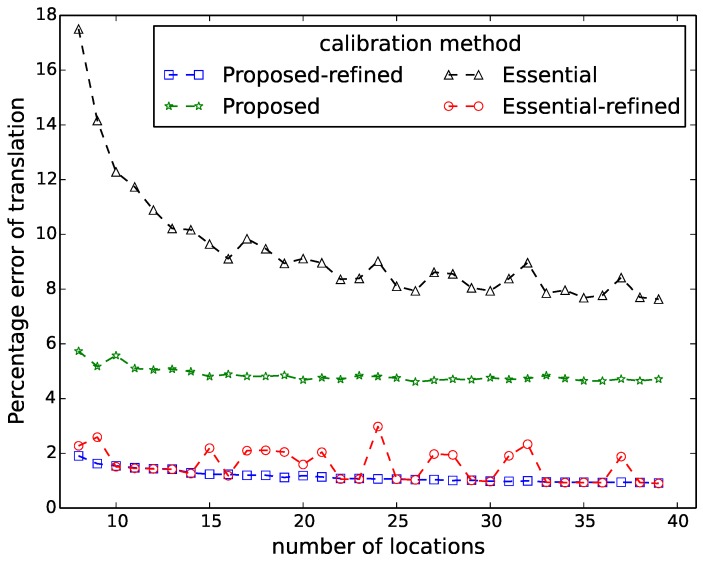
Relative translation error comparison between our method (proposed) and the method of Hödlmoser *et al.* [17] (essential) using different numbers of locations. Each location yields two calibration samples (head and feet).

**Figure 10:**
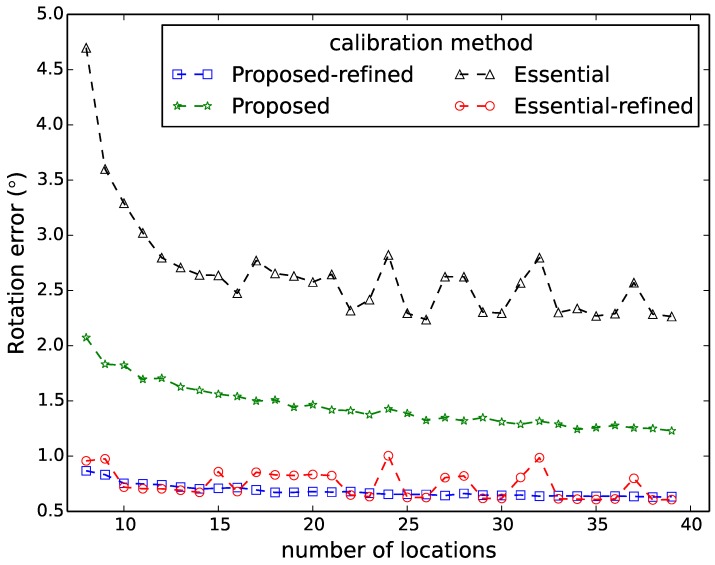
Rotation error comparison between our method (proposed) and the method of Hödlmoser *et al.* [17] (essential) using different numbers of locations. Each location yields two calibration samples (head and feet).

Figure 6, Figure 7, Figure 8, Figure 9 and Figure 10 show the comparison in terms of the projection error, the triangulation error, the reprojection error, the relative translation error and the rotation error, respectively. It can be observed from these five figures that the accuracy of the method of Hödlmoser *et al.* [17] improves by increasing the number (from eight to 39) of locations of the person. In contrast, our method can already provide the same accurate result with only eight locations of the person.

The triangulation error, the projection error, the reprojection error, the relative rotation error and the rotation error of the method of Hödlmoser *et al.* [17] are quite large when the number of locations is low (N<15). We can also observe that the refinement method can indeed improve the performance of both methods. The accuracy of both methods is almost the same after refinement, but our method provides a more accurate initial estimation of the extrinsic parameters.

### 7.2. Calibration When the Person Runs along a Straight Line

We also calibrated another camera network that is used for human motion analysis. This network has four cameras, three of which are mounted at a height of about 3 m, and one camera was mounted at a height of around 2 m. The resolution of all cameras is 780 by 580 pixels. These cameras were intrinsically calibrated using the method of Zhang [5]. In the experiments, a person ran along a straight line, which is the degenerate case for the method based on the essential matrix.

**Figure 11:**
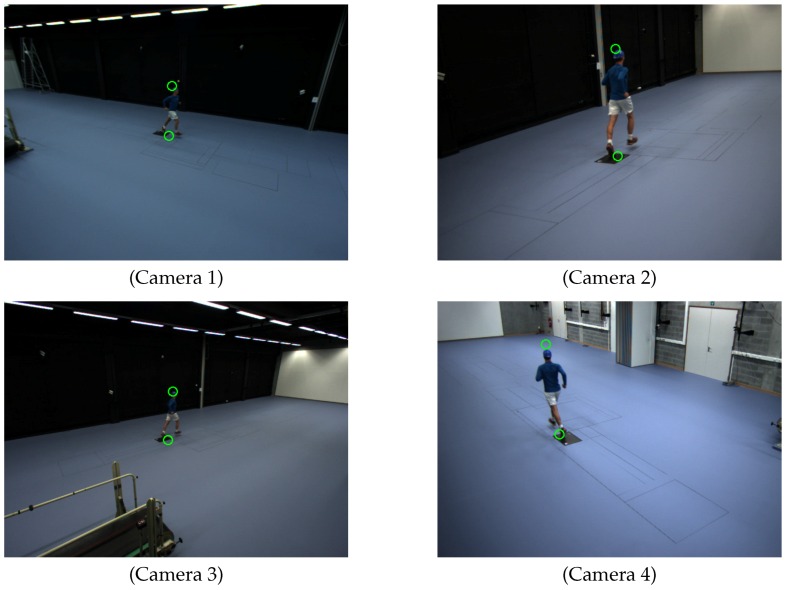
Detected feet and head positions of Camera Network 2. Green circles represent detected positions using ellipse fitting.

**Figure 12:**
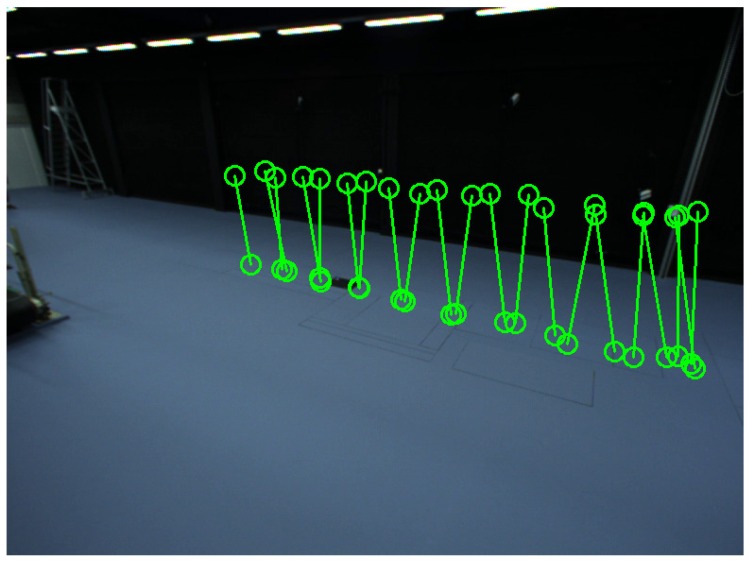
Distribution of the person’s positions for Camera Network 2. Green circles represent detected positions using ellipse fitting.

To assess the robustness to the number (*N*) of locations of the person, we repeated the calibration procedure 100 times for each *N*, for various sets of locations, randomly selected from the 21 available ones. Figure 11 shows the detected head and feet positions of the person in each scene. We detected the head and feet positions when the person was at 21 places in the common view of all cameras. Figure 12 shows the distributions of the person’s positions. We also captured four alignment samples for alignment with the predefined world coordinate system.

**Figure 13:**
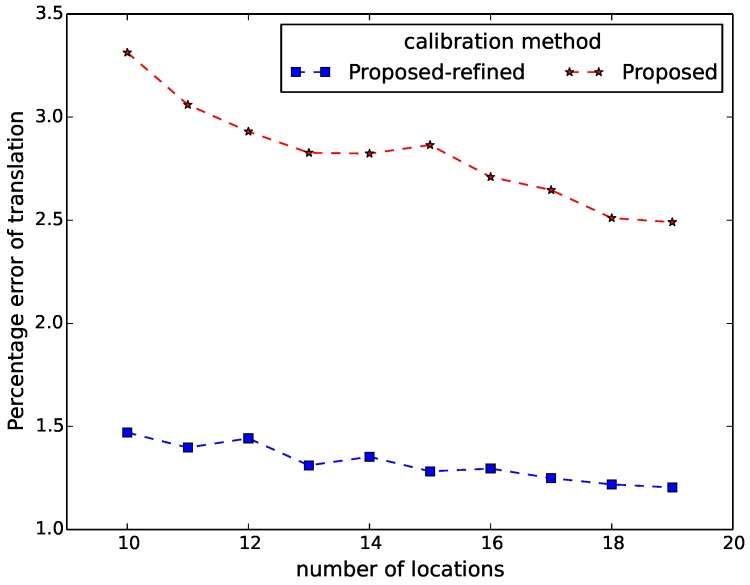
Relative translation error of our method (proposed) and the method after refinement (proposed-refined) using different numbers of locations. Each location yields two calibration samples (head and feet).

**Figure 14:**
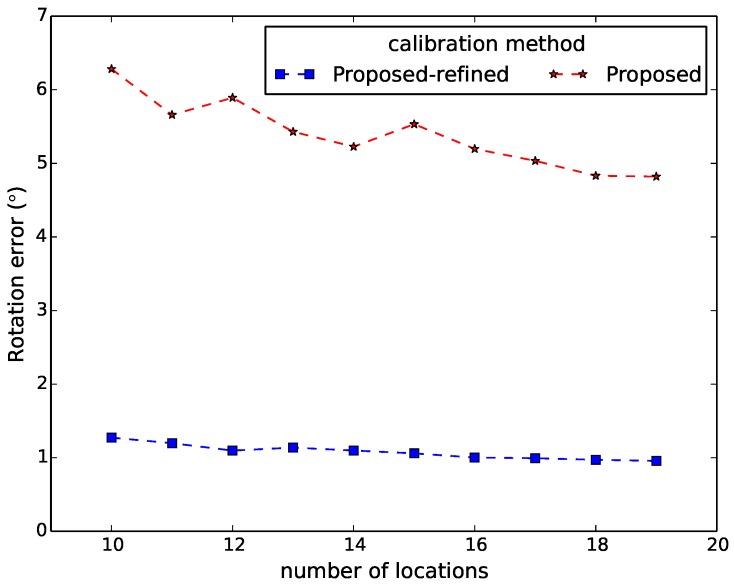
Relative rotation error of our method (proposed) and the method after refinement (proposed-refined) using different numbers of locations. Each location yields two calibration samples (head and feet).

For evaluation, we also performed extrinsic calibration using the classical method of [1]. We used the estimated extrinsic parameters from this method as the ground truth to calculate the relative rotation and translation error of our method. Figure 13 and Figure 14 display the relative translation and rotation error of our method and the method after refinement, respectively. They indicate that the proposed method provides accurate estimation of the extrinsic parameters, as the relative translation error and the rotation errors of our method after refinement are around 1.3% and $1.2^{\circ }$, respectively.

### 7.3. Calibration with Multiple Pedestrians

In the aforementioned experiments, we did calibration based on multiple observations of a single pedestrian. Our method can also be applied to the case where multiple pedestrians are in the view of each camera. We did calibration for a public dataset (EPFL-campus) from multi-camera pedestrians video dataset [44]. There are two sequences, which were shot outside on a campus with three DV cameras. The camera calibration using the Tsai calibration method [1] was provided in their dataset. We used the intrinsic calibration parameters they provide as known parameters and estimated the extrinsic parameters using our method.

**Figure 15:**
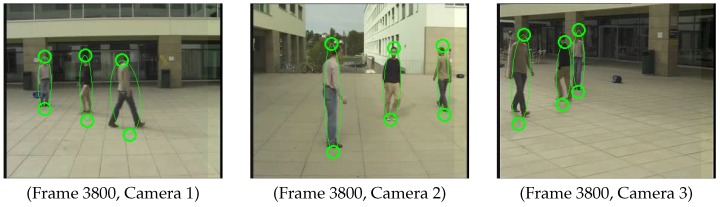
Detected feet and head positions of Camera Network 3. Green circles represent detected positions using ellipse fitting.

In order to show that our method can be applied to frame per frame online calibration, we arbitrarily selected a single frame (Frame 3800) from each camera for the second sequence, where three pedestrians (we require at least two) are in the scene, which can be seen from Figure 15. In total, there were three pairs (head and feet) of calibration samples, which means that the method of Hödlmoser *et al.* [17] is not applicable due to the lack of enough calibration samples (the method requires at least four). We manually annotated the correspondence of different persons in different camera views. We assumed an average height of those three pedestrians, since we do not know the real height of each person. For alignment with the predefined world coordinate system, we generated six alignment samples by projecting six points (with known 3D position w.r.t. the predefined world coordinate system) to each camera view, with the provided camera calibration.

As we did not have evaluation data for this network, we used the extrinsic calibration that they provided as ground truth. Then, we calculate the relative translation error and rotation error of our method. Table 4 shows the relative translation error and rotation error of each camera. We conclude that our method still provides a good estimation of the extrinsic parameters by taking only one frame from each camera.

## 8. Conclusions

In this paper, we presented a simple and robust method to compute the 3D positions of the head and feet of a person w.r.t. the camera coordinate system, which makes our method applicable for the co-planar case. We developed a RANSAC-based orthogonal Procrustes approach for pairwise calibration, which makes our method robust against outliers. Finally, an optimization routine jointly refines the extrinsic parameters for all cameras.

Compared to the method that uses classical epipolar geometry [18] to estimate extrinsic parameters, our method can provide more accurate and stable estimation of extrinsic parameters especially when there are not many observations of the person. This is quite important especially when the common view of all cameras is limited. The most important benefit of our approach is that it can still work when the person walks or even runs along a straight line, which is common in real-life situations. The method based on the essential matrix fails in this case because it cannot handle co-planar data. To the best of our knowledge, no work exists that deals with extrinsic camera calibration for this specific scenario. Our method can be applied to any other objects that can provide multiple parallel line segments of the same height. We can also provide scale information assuming the known height of the person. The scale information would allow us to provide more information about the monitored pedestrians, such as the speed of the person and walking distance of the person during a certain time period.

Our method can be applied to the case where multiple pedestrians (at least two) are in the scene. In that case, we can do online calibration (frame by frame) assuming an average height of all pedestrians. The constraint is that we need to manually annotate the correspondences between different cameras for different pedestrians. We will investigate relaxing this constraint in our future work.

## Figures and Tables

**Table 1:** List of variables.

Variables	Definition
*i*, *k*	Index for frames and cameras, respectively
$R^{\left(k\right)}$, $c^{\left(k\right)}$	Rotation and translation parameters for camera *k*
*h*	Height of the person
${\tilde{u}}_{h}^{\left(k\right)}\left(i\right)$, ${\tilde{x}}_{h}^{\left(k\right)}\left(i\right)$, $Z_{h}^{\left(k\right)}\left(i\right)$, $r_{h}^{\left(k\right)}\left(i\right)$	Image position, normalized image position, *Z* coordinate and 3D position of the head of a person, respectively, for camera *k*
${\tilde{u}}_{f}^{\left(k\right)}\left(i\right)$, ${\tilde{x}}_{f}^{\left(k\right)}\left(i\right)$, $Z_{f}^{\left(k\right)}\left(i\right)$, $r_{f}^{\left(k\right)}\left(i\right)$	Image position, normalized image position, *Z* coordinate and 3D position of the feet of a person, respectively, for camera *k*
$m^{\left(k\right)}\left(i\right)$	Cross product of ${\tilde{x}}_{h}^{\left(k\right)}\left(i\right)$ and ${\tilde{x}}_{f}^{\left(k\right)}\left(i\right)$ for camera *k*
$e_{z}^{\left(k\right)}$	Unit vector of the center line of a person w.r.t camera *k*
$\bar{r^{\left(k\right)}}$	Centroid of all 3D positions of the head and feet w.r.t camera *k*

**Table 2:** Comparison of robustness against the uncertainty of detecting the head and feet in the image between our method (proposed) and the method of Hödlmoser *et al.* [17] (essential). The numbers are listed as “a/s”, where a is the average and s is the standard deviation over 100 experiments and 2 sets of techniques for detecting the head and feet. The number of locations is 8; each location yields 2 calibration samples (head and feet).

	Proposed-Refined	Proposed	Essential	Essential-Refined
Triangulation error (cm)	**1.9/0.8**	5.4/1.5	10.0/7.6	2.0/1.7
Projection error (pixel)	**4.6/0.6**	8.2/2.2	33.2/20.9	4.8/1.8
Reprojection error (pixel)	**4.4/0.6**	7.1/2.1	32.1/20.9	4.6/1.6
Rotation error (${}^{\circ }$)	**0.9/0.2**	2.2/0.6	4.1/3.8	1.1/2.2
Relative translation error	**1.9%/0.8%**	6.6%/1.8%	14.6%/9.9%	2.6%/7.4%

**Table 3:** Success percentages (number of successful estimations within 1000 experiments) of our method (proposed) and the method of Hödlmoser *et al.* [17] (essential). The first row represents the number of locations, and the first column gives the methods used for comparison. Each location yields 2 calibration samples (head and feet).

	2	3	4	5	6	7
Essential	NA	NA	35.3%	76.1%	93.6%	97.9%
Proposed	63.3%	91.8%	97.8%	99.7%	99.9%	100%

**Table 4:** Rotation and relative translation error of our method after refinement for each camera.

	Camera 1	Camera 2	Camera 3
Rotation error (${}^{\circ }$)	2.0	5.1	3.1
Relative translation error	5.7%	1.5%	0.8%

## Abbreviations

The following abbreviations are used in this manuscript:

2D: Two-dimensional
3D: Three-dimensional
VSS: Visual surveillance system
DLT: Direct linear transformation
RANSAC: Random sample consensus
SVD: Singular value decomposition
